# The association between experience of hospital accreditation and nurses’ perception of patient safety culture in South Korean general hospitals: a cross-sectional study

**DOI:** 10.1186/s12912-021-00708-x

**Published:** 2021-10-12

**Authors:** M. R. Kwan, H. J. Seo, S. J. Lee

**Affiliations:** 1 Department of Nursing, Gwangju Veterans Hospital, Gwangju, 62284 South Korea; 2 College of Nursing, Chungnam National University, 266 Munhwa-ro, Jung-gu, Daejeon, 301-747 South Korea; 3 College of Nursing, Korea University, Seoul, 02841 South Korea

**Keywords:** Patient safety culture, Accreditation, Hospital, Association

## Abstract

**Background:**

Hospital accreditation programs can positively affect nurses’ perceptions of patient safety culture. However, no previous research has identified the association between experience of hospital accreditation and nurses’ perception of patient safety culture in general hospitals. This study aims to examine 1) the level of perception of each area of patient safety culture in nurses working in general hospitals and 2) the relationship between experience of hospital accreditation and nurses’ overall perceptions of safety in Korean general hospitals.

**Methods:**

A cross-sectional survey design was used, with a convenience sample of 310 nurses from six general hospitals. Nurses were asked to complete the self-reported Korean version of the Hospital Survey on Patient Safety Culture and the experience of hospital accreditation. A hierarchical multiple regression analysis was used to examine the associations between hospital accreditation experience and perception of patient safety culture.

**Results:**

The patient safety composites with the highest positive response were the frequency of events reported (90.6) and supervisor/manager expectations promoting patient safety (69.4%). The composites with the lowest scores were non-punitive responses to errors (22.9%) and organizational learning/continuous improvement (35.5%). Hierarchical multiple regression analysis showed that the experience of hospital accreditation had a very small increase on overall perceptions of safety (*β* = 0.097, *p* = 0.023).

**Conclusions:**

This study found that general hospital nurses’ experience of hospital accreditation had very weak relationship with their overall perceptions of patient safety. Therefore, a longitudinal study is needed to confirm the influence of hospital accreditation on nurses’ patient safety culture in general hospitals.

## Background

According to a report in 2000 by the US National Institute of Medicine, 44,000–98,000 patients die from patient safety incidents annually [1]. In Korea, approximately 17,000 patients die from preventable patient safety events annually, accounting for 43.5% of medical accident deaths [2]. Recently, patient safety has received increasing attention, resulting in an increased number of quality improvement programs, and heightened regulatory requirements for healthcare organizations [3]. Accreditation is an external review program that assesses how employees perform against established standards [4]. Accreditation, as used in over 70 countries [5], is compelling, helps staff strengthen patient safety, and improves the quality of care [6].

As part of a national effort to improve healthcare quality, the Accreditation Program for Healthcare Organizations in Korea has been in effect since 2002 for general hospitals and those with more than 300 beds. With an amendment to medical law in June 2010, the assessment of medical institutions has shifted from compulsory to a self-regulated certification system; since June 2012, in particular, small- and medium-sized non-tertiary hospitals have shown more active participation [7].

A recent review of the perceptions held by various healthcare personnel regarding accreditation found that hospital accreditation affected the quality of care through improvements in employee engagement, management quality, and outcome quality [8]. By treating safety management and patient identification as important factors in preventing patient safety incidents (in accident-prone areas in the course of care), the certification system had an impact on nurses’ patient safety awareness [9]. Moreover, previous studies reported that nurses’ performance and the quality of nursing services improved through the establishment of specialized processes and work manuals, which, in turn, was reported to have a positive effect on their job satisfaction [10].

Nurses work closely with patients and are most sensitive to patient safety incidents. As such, it is necessary to improve and strengthen patient safety awareness through repeated training and education programs to ensure that patients and caretakers receive safe treatment. The creation of a patient safety culture requires hospitals’ active participation in accreditation programs. Therefore, more research on nurses’ perceptions is necessary to determine the impact of hospital accreditation on the quality of care [8].

In a recent study, research on patient safety culture has showed that patient safety culture is associated with the intention to report near-miss [11] and adverse events [12]. A qualitative study on hospital accreditation experience reported that accreditation programs affected the priorities of management (e.g., working on the documentation) and improved organizational structure during preparation for accreditation [13]. Although few Korean studies have reported that there is a positive correlation between perceptions of patient safety culture and safety nursing activities [14, 15], there are no studies on the association between hospital accreditation experience and nurses’ perceptions of patient safety culture. Therefore, this study intends to examine the association between hospital accreditation experience and nurses’ perceptions of patient safety culture in general hospitals.

The purposes of this study are as follows:

1. Assess the level of perception of each area of patient safety culture in nurses working in general hospitals.
2. Identify the relationship between experience of hospital accreditation and nurses’ overall perceptions of safety as a sum of four items:

(Question 10) *It is just a matter of luck that more serious mistakes do not occur around here.*

(Question 15) *Patient safety is never compromised to get more work done.*

(Question 17) *We have patient safety problems in this unit.*

(Question 18) *Our procedures and systems are effective in preventing errors.*

## Methods

### Study design

This cross-sectional study aimed to investigate the relationship between the experience of hospital accreditation and patient safety culture in nurses in general hospitals.

### Setting, sampling, and participants

Six general hospitals were selected using convenience sampling; three of these institutions had obtained hospital accreditation, while the other three did not. To increase the comparability of groups, we selected the study hospitals based on matching methods including hospital type (general hospital), size (two hospitals under each of the three categories based on bed capacity of 100–199, 200–299, 300 or more, respectively), and location (in Gwangju city). Subsequently, nurses working for the selected hospitals were recruited for participation. The target population included all registered nurses who were able to write and read Korean and had at least one year of experience in their respective units, to ensure that they were familiar with the hospital’s policy and the rules relevant to hospital accreditation [16].

The sample size was calculated using G*Power 3.1.9.7, which revealed that a minimum of 311 participants was needed to perform a regression analysis with a small effect size of 0.02, significance level of 0.05, statistical power of 0.80, and eight predictors (age, education level, working unit/area, work experience, job position, hospital size, patient safety culture area, and accreditation status). To account for incomplete responses, questionnaires were distributed to 330 participants. A total of 310 questionnaires were collected, for a response rate of 94%.

### Measurement tools and outcome measures

The study used the Korean version of the Hospital Survey on Patient Safety Culture (HSOPSC) [17]. The HSOPSC is composed of 44 items covering 12 composites of patient safety culture [18]. This study used the Korean version of the HSOPSC, as verified by Kim et al. [17] (with permission from the original author in June 2014). The researchers from diverse professional backgrounds comprehensively evaluated the concepts in this study and the purpose and relevance of each questionnaire to confirm whether the ratio of different types of content distribution was appropriate. For the Korean version of the study, Cronbach’s coefficient (α), which represents the internal consistency of the tool, was 0.905 [17].

Items were scored on a five-point Likert scale. The percentage of positive responses for each item was subsequently calculated; negatively worded items were reversed when computing the percentage of positive responses. We followed the HSOPSC user’s guide [18] for data analysis, to allow for benchmarking results. Positive responses in positively worded survey items were “agree/strongly agree” or “most of the time/always.”

The outcome variable was overall perceptions of safety (as a sum of four items). In this study, the internal consistency of the instrument was measured using Cronbach’s α. The highest value (0.85) was for the frequency of reported events, and the lowest value (0.22) was for the non-punitive response to error. Two of the composites (Cronbach’s α of non-punitive response to error = 0.22, Cronbach’s α of Staffing = 0.38) were measured at a level below the acceptable level of α ≥ 0.6 defined in the HSOPSC user’s guide [18].

The survey tool captured the following demographic characteristics of the participants: current work experience (1–5, 6–10, 11–15, 16–20, 21 years and above), experience with a hospital accreditation system (yes/no), education level (3-year college, 4-year university, graduate school or more), working unit/area (medical ward, surgical ward, pediatric ward, dialysis room, intensive care unit, operating room, others), age, position (general nurse, charge nurse, head nurse or higher), and hospital size (100–199, 200–299, 300 or more).

### Data collection

The data for this study were collected between July and September 2014, using structured self-report questionnaires distributed to nurses working at six general hospitals. Prior to data collection, the researchers visited the hospitals’ nursing department to explain the study’s purpose and obtain approval for data collection.

### Data analysis

The participants’ general characteristics and experience of hospital accreditation system were presented as frequency and percentage values, along with the mean and standard deviation of the recognition of patient safety culture in each area. And then, the response rate for each survey question was calculated as follows: positive (4–5 points on a 5-point Likert scale), neutral (3 points on a 5-point Likert scale), and negative (1–2 points on a 5-point Likert scale). Lastly, differences in the perception of patient safety culture based on demographic factors and experience of hospital accreditation (with or without certification) were analyzed using multiple comparisons between the independent two-sample test or one-way ANOVA and Duncan tests. Fourth, a hierarchical multiple regression analysis was conducted to investigate the association of general characteristics, patient safety culture area, and experience of medical institution certification on patient safety culture outcomes. The variance inflation factor (VIF) was used to determine whether there was a multicollinearity problem (which is a correlation between independent variables in the regression analysis). The histogram and plot of residuals were examined to review the linearity and normality of the regression analysis, which is the assumption in the regression analysis. The analysis was conducted using SPSS IBM 20.0, and the level of statistical significance was set at 0.05 for two-tailed tests.

### Ethical considerations

The present study was approved by the Institutional Review Board (IRB No. IRB 2014-7th-3) of Gwangju Veterans Hospital and was conducted in accordance with the Declaration of Helsinki.

## Results

### Respondents’ characteristics

The descriptive statistics for the study respondents are presented in Table 1. The size distribution of the hospitals was as follows: 21.0% had 100–199 beds, 22.6% had 200–299 beds, and 56.5% had 300 beds or more. Respondents’ average age was 30.99 (standard deviation = 7.65). The educational attainment of a majority of the participants (57.4%) was at the diploma level, and 67.1% of respondents worked in their current department for 1–5 years. Among the respondents, 63.2% did not have experience in accreditation evaluations, 28.4% currently worked in surgical units, 22.3% were in the medical unit, and 22.3% were in the intensive care unit or operating room.

**Table 1:** General characteristics of respondents (*N* = 310)

		N	%	M ± SD	Range
Age	≤24	56	18.1	30.99 ± 7.65	22–55
	25–29	109	35.2		
	30–34	65	21.0		
	35–39	37	11.9		
	≥40	43	13.9		
Education	Diploma	178	57.4	–	–
	Bachelor	111	35.8		
	Master	21	6.8		
Working unit/area	Internal medicine	69	22.3	–	–
	General surgery	88	28.4		
	OBGY/PED	42	13.5		
	ER/Hemo	42	13.6		
	ICU/OR	69	22.3		
Current work experience	1–5	208	67.1	–	–
	6–10	61	19.7		
	11–15	23	7.4		
	≥ 16	18	5.8		
Hospital size	100–199	65	21.0	–	–
	200–299	70	22.6		
	≥ 300	175	56.5		
Position	Staff nurse	263	84.8	–	–
	Charge nurse	21	6.8		
	Head nurse	26	8.4		
Experience of accreditation	No	196	63.2	–	–
	Yes	114	36.8		

*OBGY/PED* Obstetrics & Gynecology, Pediatrics

*ER/Hemo* Emergency Room, Hemodialysis Room

*ICU/OR* Intensive Care Unit, Operating Room

### Research question 1: the level of perception among nurses at general hospitals against each area of patient safety culture

#### Patient safety culture composite-level average percentage-positive scores

In units, “Supervisor/manager expectations and promoting patient safety” showed the highest rate of positive responses at 69.4%, followed by “Staffing” at 61.5%, and “Teamwork within units” at 51.8%. However, “Non-punitive response to error” had the lowest rate at 22.9%, with slightly better outcomes for “Organizational learning/continuous improvement” at 35.5%, and “Communication openness” at 43.1%.

In hospitals, “teamwork across hospital units” showed the highest rate of positive responses (50.6%), followed by “management support for patient safety” (43.6%), and “handoffs and transitions” (43.5%) (Table 2).

**Table 2:** Survey composites and items’ positive scores (*N* = 310)

Composites and survey items	Average percentage-positive response
**Comparison of perceptions regarding patient safety culture in units**	
**Supervisor/manager expectations and promoting patient safety (Cronbach’s α = 0.68)**	**69.4**
My supervisor/manager praises staff when a job is done according to established patient safety procedures	59.7
My supervisor/manager seriously considers staff suggestions for improving patient safety	67.9
Whenever pressure builds, my supervisor/manager wants us to work faster, even if it means taking shortcuts(R)	63.4
My supervisor/manager overlooks patient safety problems that recur(R)	86.4
**Organizational learning/continuous improvement (**Cronbach’s α = 0.41)	**35.5**
We are actively improving patient safety	17.1
Mistakes have led to positive changes here	40.5
After we make changes to improve patient safety, we evaluate their effectiveness	49.0
**Teamwork within units (**Cronbach’s α = 0.53)	**51.8**
People support each other in terms of work in this unit	11.9
When a lot of work needs to be done quickly, we work together as a team to get it done	65.4
In this unit, people treat each other with respect	77.1
When one area in this unit gets really busy, others help out	52.7
**Nonpunitive response to errors (**Cronbach’s α = 0.22)	**22.9**
Staff feel like their mistakes are held against them(R)	20.0
When an event is reported, it feels like the person is being reported, not the problem(R)	18.5
Staff worry that mistakes are recorded in their file	28.9
**Staffing (Cronbach’s α = 0.38)**	**61.5**
We have enough staff to handle the workload	88.0
Staff in this unit work long hours, which might affect patient care	53.2
We use more agency/temporary staff than is best patient care	70.2
When work is in “crisis mode,” we try to do too much, too quickly	33.2
**Communication openness (Cronbach’s α = 0.63)**	**43.1**
Staff freely speak up if they see something that may negatively affect patient care	45.4
Staff feel free to question the decisions or actions of those with more authority	36.1
Staff are afraid to ask questions when something does not feel right(R)	48.0
**Feedback and communications about errors (Cronbach’s α = 0.68)**	**66.3**
We are given feedback about changes made on the basis of event reports	61.7
We are informed about errors that happen in this unit	73.8
We discuss ways to prevent errors from recurring in this unit	63.4
**Comparison of perceptions regarding patient safety culture in hospital**	
**Management support for patient safety (Cronbach’s α = 0.56)**	**43.6**
Hospital management provides a work climate that promotes patient safety	56.6
Hospital management show that patient safety is a top priority	40.9
Hospital management is interested in patient safety only after an adverse event (R)	33.2
**Teamwork across hospital units (Cronbach’s α = 0.55)**	**50.6**
People support one another in terms of work in this unit(R)	54.8
When a lot of work needs to be done quickly, we work together as a team to get it done	43.4
In this unit, people treat each other with respect(R)	51.3
When one area in this unit gets really busy, others help out	52.9
**Handoffs and transitions (Cronbach’s α = 0.74)**	**43.5**
Important patient care information is often lost during shift changes(R)	38.7
Important patient care information is often lost during shift changes(R)	56.3
Problems often occur in the exchange of information across hospital units(R)	25.6
Shift changes are problematic for patients in this hospital(R)	58.2
**Perception and frequency of error reports on overall patient safety**	
**Overall perception of safety (Cronbach’s α = 0.54)**	**55.7**
It is just a matter of luck that more serious mistakes do not occur around here(R)	73.6
Patient safety is never compromised to get more work done	51.4
We have patient safety problems in this unit(R)	55.6
Our procedures and systems are effective in preventing errors	42.0
**Frequency of events reported (Cronbach’s α = 0.85)**	**90.6**
Events caught and corrected before it can affect the patient	92.3
Events with no potential to harm the patient	90.6
Events that could harm the patient but did not	88.5
**Hospital patient safety grade (1 item)**	**36.4**

*R* Reverse coding item

### Research question 2: the relationship between experience of hospital accreditation and nurses’ overall perceptions of safety

#### Differences in perceptions of patient safety culture according to respondents’ characteristics

Overall perceptions of safety, according to general characteristics, were statistically significant for the variables of job position (F = 6.42, *p* = 0.002) and current work experience (F = 3.27, *p* = 0.02). Head nurses had greater overall patient safety awareness than general nurses and chief nurses, and those with work experience of 16 years or more had higher patient safety culture awareness (at a statistically significant level) than those with 1–5, 6–10, and 11–15 years of experience (Table 3).

**Table 3:** Overall perceptions of safety according to general characteristics (*N* = 310)

Characteristics	Categories	Mean ± SD	Overall Perception of Safety		
			F-value	***p***-value	Duncan’s multiple comparison
Age	≤24	14.14 ± 2.31	1.66	1.160	
	25–29	13.58 ± 2.07			
	30–34	13.68 ± 2.61			
	35–39	13.54 ± 3.28			
	≥40	14.58 ± 2.58			
Education	Diploma	13.82 ± 2.46	1.65	0.194	
	Bachelors	13.69 ± 2.51			
	Masters	14.76 ± 2.44			
Working unit/area	Internal Medicine	13.75 ± 2.30	1.47	0.212	
	General Surgery	13.50 ± 2.55			
	PED/OBGY	14.00 ± 3.03			
	Hemo/ER	13.61 ± 2.31			
	ICU/OR	14.40 ± 2.25			
Current work experience	1–5^a^	13.77 ± 2.45	3.27	0.02*	a,b,c < d
	6–10^b^	13.73 ± 2.25			
	11–15^c^	13.39 ± 2.75			
	≥ 16^d^	15.55 ± 2.79			
Hospital size	100–199	13.78 ± 3.03	1.21	0.300	
	200–299	14.24 ± 2.07			
	≥ 300	13.70 ± 2.40			
Position	Staff nurse^a^	13.66 ± 2.45	6.42	0.002**	a,b < c
	Charge nurse^b^	14.00 ± 2.49			
	Head nurse^c^	15.46 ± 2.31			

* *p* < 0.05, ** *p* < 0.01

*OBGY/PED* Obstetrics & Gynecology, Pediatrics, *ER/Hemo* Emergency Room, Hemodialysis Room, *ICU/OR* Intensive Care Unit, Operating Room

#### Association of the medical institution accreditation system and patient safety culture

To examine the factors affecting the overall perceptions of safety (as a sum of four items), hierarchical multiple regression analysis was performed using the following as independent variables: the general characteristics of the respondents (work experience, position), patient safety culture area (department environment, direct manager, communication, frequency of reports, hospital), and experience with medical institution accreditation. Verification of the assumption of the regression analysis for the independent variables showed that the tolerance was between 0.19 and 0.97, and the VIF was 1.02 to 5.18; thus, there was no multicollinearity. The residual analysis showed no specific pattern of increasing or decreasing residuals on the residual plot, thus satisfying the assumption of homoscedasticity .

The overall perceptions of safety were statistically significant (R^2^ = 0.52, F(*p*) = 3.36(0.006)] among head nurses or higher (*β* = 0.159, *p* = 0.009) in Model 1; work environment (*β* = 0.589, *p = < 0.001*) and hospital management and support for patient safety (*β* = 0.261, *p* = < 0.001) in patient safety culture, which was added to Model 2, increased the explanatory power of the model at a statistically significant level [∆R^2^ = 0.423, F(*p*) = 26.59(< 0.001)]. In Model 3, where components of patient safety culture were added, the adjusted R^2^ was 0.007 (*p* < 0.001), showing very small increase in “overall perception of safety,” thereby making it a predictor (*β* = 0.097) (Table 4). In other words, the dependent variable (overall perception of patient safety) consisted of 4 items on a 5-point Likert scale, with a total score ranging from 4 to 20 points. For the independent variable (experience of hospital accreditation), the beta coefficient was 0.097, indicating that the experience of hospital accreditation was associated with an increase of 1.94 points (20 points X0.097) in the dependent variable. Overall, there was a very small increase in the dependent variable ‘experience of hospital accreditation’ by approximately 2 points (10%).

**Table 4:** Factors affecting overall perceptions of safety (*N* = 310)

Variable		Overall perceptions of safety (sum)					
		Model 1		Model 2		Model 3	
		β	***p***	β	***p***	β	***p***
Current work experience							
	1–5	Referent group					
	6–10	−0.214	0.085	0.063	0.515	0.043	0.652
	11–15	−0.183	0.107	0.023	0.790	0.009	0.917
	≥16	−0.163	0.056	0.019	0.780	0.008	0.909
Position							
	Staff nurse	Referent group					
	Charge nurse	0.028	0.628	−0.015	0.729	−0.014	0.758
	Head nurse	0.159	0.009**	0.075	0.110	0.070	0.132
Patient safety culture	Working environment			0.589	<.001**	0.587	<.001**
	Supervisor/manager			0.003	0.954	−0.002	0.971
	Communication			−0.087	0.140	−0.077	0.187
	Frequency of events reported			−0.047	0.281	−0.044	0.307
	hospital management and support for patient safety			0.261	<.001**	0.267	<.001**
Experience of accreditation	Yes vs. No					0.097	0.023*
R^2^		0.052		0.477		0.486	
adj R^2^		0.036		0.459		0.466	
(⊿R^2^)		0.423			0.007		
F(p)		3.36(0.006**)		26.59(<.001**)		24.99(<.001**)	

* *p* < 0.05, ** *p* < 0.01

## Discussion

The findings revealed that the environment of the working department, hospital management and support for patient safety, and nurses’ experience of accreditation were related to the overall perception of safety. In general hospital settings, the perception of patient safety culture among nurses was as follows: the highest positive response rate for patient safety culture dimensions was “Frequency of events reported (90.6%),” which was higher than the results of the Agency for Healthcare Research and Quality in the United States of America (67%), Lebanon (68.2%), and Saudi Arabia (64.3%) [16]. This indicates Korean nurses’ high perception that nurses should report near-miss events when no actual errors have occurred. The second-highest positive response rate was “Supervisor/manager expectations and promoting patient safety (69.4%).” This is lower than the results for the United States (87%) and Saudi Arabia (70%), but higher than those for Palestine (56%) and Lebanon (66.4%) [19]. Accreditation performance was reported to be positively correlated with leadership and organizational culture [20]. This result is supported by another study that found that leadership, quality management, commitment, and support are all predictors of quality improvement during and after the accreditation process [21].

The results for “Teamwork within units (51.84%)” were perceived to be higher than those for “Teamwork across hospital units (50.6%),” and were similar to those in Palestine (44–74%), Lebanon (56–82.3%), and the United States (50–84%), which is consistent with another study on nurses in general hospitals in Korea [22]. Hospitals have diverse health care staff, specialized organizations, complex function-sharing systems, and medical services which are characterized by close interrelationships among healthcare staff. Thus, various members of hospital departments must cooperate with each other to achieve this goal [23]. In addition, a multidisciplinary team with good communication, in which doctors participate actively, can promote the continuous implementation of care quality improvement [24]. Therefore, cooperation between departments within the hospital needs to be improved to create a patient safety culture.

The areas with the lowest positive rates were “Non-punitive response to errors (23%)” and “Organizational learning/continuous improvement (35.6%).” Non-punitive responses to errors ratings were similar to those in Palestine (17%), Lebanon (24.3%), Saudi Arabia (22%), and the United States (45%). These results suggest that there is a need to foster an organizational culture that promotes tolerance for mistakes and demands individual responsibility for medical errors [19]. This is consistent with the findings of Korean studies showing that Korea has the lowest rate of punitive responses on the perception of disclosure of patient safety incidents [25]. A culture of blaming in the event of a patient safety incident decreases the willingness to report and resolve problems voluntarily when an incident arises, hindering awareness about problems occurring in the hospital and potential solutions. Blaming not only interferes with the opportunity to prevent recurrence of patient safety incidents but also makes it difficult for hospitals to establish a patient safety culture [26].

Establishing a system within the hospital environment to identify the causes of patient safety incidents can reduce the recurrence of errors [17]. To establish a patient safety culture, medical institutions must work hard to establish patient safety management procedures or systems to reduce patient safety incidents. Forming an organization’s safety culture, including activities for systematic learning and improvement (such as measuring the effects after an attempted change) takes effort and attention; mistakes lead to positive changes, and cooperation among departments in the course of providing medical services to hospitals must be emphasized [25].

In this study, the “overall perception of safety” was 55.7%, which was lower than that in the United States (73%), Palestine (62%), Lebanon (78.3%), and Saudi Arabia (87%). The positive response rate for “Patient safety rating of hospital” was 36.4%, which was lower than that in the United States (76%), Palestine (63.5%), Lebanon (73.4%), and Saudi Arabia (43.0%) [18]. These results show that hospitals exhibit passive behavior rather than active efforts to build a safety culture within the organization; patient safety culture awareness at the hospital level needs improvement.

Hierarchical multiple regression analysis was conducted to investigate factors that influence “overall perception of patient safety,” showing that head nurses or higher (*p* = 0.009) among the positions in Model 1, the work environment in patient safety culture areas (*p* < 0.001), added to Model 2; and hospital management and support for patient safety (*P* < 0.001) increased the explanatory power of the model at a statistically significant level; hospital accreditation experience, added to Model 3, had a significant association on patient safety culture. These findings are similar to those conducted in Lebanese nurses [27], wherein accreditation was found to have a positive effect on numerous patient safety culture predictors (β = 1.041, (*p* < 0.001). In a survey of university hospital of Turkey, hospital accreditation has a positive impact on quality results in nurses (β =0.541, (p < 0.001)) [28].

However, the results of a Jordanian study [16] reported that “experience with hospital accreditation” did not have a positive association with patient safety culture. In another study, benefits of accreditation was not associated with quality results in tertiary hospital nurses (β = 0.005 ((95% CI = − 0.038 to 0.048)) [29]. Moreover, a systematic review of accreditation [30] did not find evidence supporting the view that accreditation and certification of hospitals were associated with quantifiable changes in quality of care, such as those measured by quality metrics. Consequently, the strategies that hospitals should pursue to improve patient safety and the results related to certification and accreditation components remain unclear.

In this study, the experience of hospital accreditation for general hospital nurses was reported to have a very weak relationship on overall perceptions of patient safety. These findings can be interpreted as follows: the concept and perception of patient safety culture have not evolved yet, and the legitimacy of and active participation in the medical institution accreditation system may have been absent during the 1-cycle accreditation evaluation that was in progress during the data collection period. In addition, the autonomous certification system must be implemented in a top-down manner and employing human resources and a proper infrastructure to conduct evaluation. Accreditation in clinical work is bureaucratic, resource intensive, and time-consuming [31]. Therefore, health authorities need to provide incentives to medical institutions to reduce the cost burden of participation in institutional accreditation and help improve the level of patient safety and quality of care within organizations through participation at the general hospital level.

Nevertheless, the limitations of this study are as follows. This study focused on nurses at general hospitals in G city, using non-probability-based sampling; resulting in difficulty to generalize. Furthermore, cross-sectional survey studies can provide information on the association between medical institution certification experience and patient safety culture, and not on the causal relationship. In addition to the individual-level factors, such as heavy workload, lack of motivation about safety care, job satisfaction, and affective commitment [29], and organization-level factors, such as case-mix index, staffing adequacy (e.g., patient to nurse ratio), organizational priorities, incentives, financial status (e.g., infrastructure), hospitalization rate, and the availability of patient safety education, may affect patient safety culture. Therefore, further studies that consider un-identified factors that influence nurses’ overall perception of patient safety need to be conducted. Finally, although the data of our study were not relatively recent, this is the first study to identify the association between nurses’ perceptions of patient safety culture and their experience of hospital accreditation in general hospital settings. Exploring the association between these perceptions can inform strategies to improve nurses’ working environment and hospital management and support for patient safety culture.

## Conclusion

This study found that the patient safety composites with the highest positive response were the frequency of events reported, supervisor/manager expectations, promoting patient safety, and feedback and communication about errors. The composites with the lowest scores were non-punitive response to error, organizational learning/continuous improvement, and communication openness. In the hierarchical multiple regression analysis, general hospital nurses’ experience of hospital accreditation had very weak association on overall perceptions of patient safety.

### Implications for nursing management

To establish a patient safety culture, medical institutions must establish patient safety management systems. Doing so will require dedicated effort from those involved in the formation of organizations’ safety culture, including providing activities for systematic learning, training, and improvement. It is also necessary to conduct longitudinal study evaluating the impact of hospital accreditation on quality results including overall perceptions of patient safety in nurses of general hospitals.

## Data Availability

The datasets used and/or analyzed during the current study available from the corresponding author on reasonable request.

## Abbreviations

HSOPSC: Hospital Survey on Patient Safety Culture
